# Systemic lupus erythematosus-associated neutrophilic dermatoses

**DOI:** 10.1016/j.jdcr.2025.10.063

**Published:** 2025-11-13

**Authors:** Sabrina M. Saeed, Amanda G. Zhou, Jeff R. Gehlhausen, Christine J. Ko, Fotios Koumpouras, Caroline A. Nelson, Sarika Ramachandran

**Affiliations:** a Yale School of Medicine, New Haven, Connecticut; b Department of Dermatology, Yale School of Medicine, New Haven, Connecticut; c Section of Allergy, Immunology & Rheumatology, Department of Internal Medicine, Yale School of Medicine, New Haven, Connecticut

**Keywords:** cutaneous lupus erythematosus, neutrophilic dermatoses, systemic lupus erythematosus

## Introduction

Neutrophilic infiltrates in cutaneous lupus erythematosus (CLE) represent an uncommon but increasingly recognized histopathologic variant sometimes termed systemic lupus erythematosus (SLE)–associated neutrophilic dermatosis (ND).^1^^,^^2^ Unlike classic CLE, which demonstrates a lymphocytic interface dermatitis with mucin deposition, neutrophil-predominant cases are characterized by dense interstitial or perivascular neutrophilic infiltrates, often with leukocytoclasia and only subtle interface change.^1^, ^2^, ^3^, ^4^ Clinically, these lesions are heterogeneous, presenting as erythematous papules, plaques, urticarial lesions, or annular eruptions, sometimes mimicking Sweet syndrome or neutrophilic urticarial dermatosis (NUD). Histologically, the density of neutrohpils may mimic Sweet syndrome while some cases demonstrate a hybrid pattern with lupus-specific features such as vacuolar alteration and basement membrane immunoreactants as well as perivascular and sometimes interstitial neutrophils.^1^, ^2^, ^3^, ^4^

Prior research^1^^,^^2^^,^^5^ has attempted to define the clinicopathologic spectrum of SLE-associated NDs, noting that they are widely heterogeneous, with a broad spectrum of clinical and histopathologic presentations. Additionally, prognostic implications and optimal treatment strategies remain unclear. Given the potential for overlap with both classic CLE and other neutrophilic dermatoses, a clearer understanding of this entity is essential.

The present case series aims to better define the clinical and histopathologic features of SLE-associated NDs and to clarify its relationship to the wider spectrum of neutrophilic lupus lesions. To that end, all cases of SLE-associated ND included in this series were reviewed for demographic, clinical, histopathologic, and laboratory data, and the 2012 Systemic Lupus International Collaborating Clinics^6^ and 1997 American College of Rheumatology^7^ criteria for SLE were used to corroborate the diagnosis of SLE. A board-certified dermatopathologist reviewed slides from these cases to confirm histopathologic findings. A summary of these cases can be found in Table I. This study was approved by the Yale Institutional Review Board.

**Table I:** Summary of cases of SLE-associated ND

Case	Age/gender	Prior history of SLE?	Morphology/distribution	Associated symptoms	Extractable nuclear antigen (Sm, RNP, SSA, SSB, Scl70, Jo1) status	Histopathology	Treatment for SLE-associated ND
1A∗	32/F	Yes	Papules and plaques/bilateral palms	Fatigue, headache, arthralgias, angioedema	Sm+, RNP+, SSA+ SSB− Scl70 & Jo1 NT	“Sweet’s-like”–dermal neutrophilic infiltrate but without papillary edema. No interface change.	Increased dosing of systemic steroids
1B∗			Papules and plaques/widespread	Fever, headache, pruritus, arthralgias		Superficial, sparse perivascular neutrophils. No interface change.	Increased systemic steroids, increased dapsone/colchicine, antihistamines
2	28/M	Yes	Edematous papules and plaques/face and arm	Arthralgias, angioedema	Sm+, RNP+, SSA−, SSB−, Scl70−, Jo1 NT	Diffuse mild superficial and deep neutrophilic infiltrate. No interface change.	Increased colchicine, added dapsone, antihistamines, anifrolumab, and voclosporin
3	48/F	No	Annular plaques/bilateral thighs	Arthralgias, proximal weakness	Sm−, RNP−, SSA−, SSB−, Scl70−, Jo1−	Mixed dermal infiltrate of neutrophils and rare eosinophils. No interface change.	Systemic steroid taper followed by HCQ start

*F*, Female; *HCQ*, hydroxychloroquine; *ND*, neutrophilic dermatosis; *NT*, not tested; *SLE*, systemic lupus erythematosus.

∗ Case 1A = case 1 at initial presentation; Case 1B = case 1 at subsequent presentation.

## Case 1

**Fig 1:**
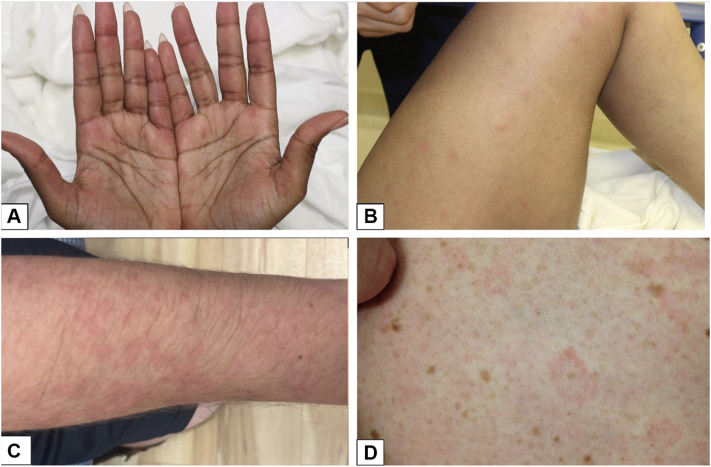
SLE-associated neutrophilic dermatosis in **(A)** case 1 at initial presentation, **(B)** case 1 at subsequent presentation, **(C)** case 2, and **(D)** case 3. *SLE*, Systemic lupus erythematosus.

**Fig 2:**
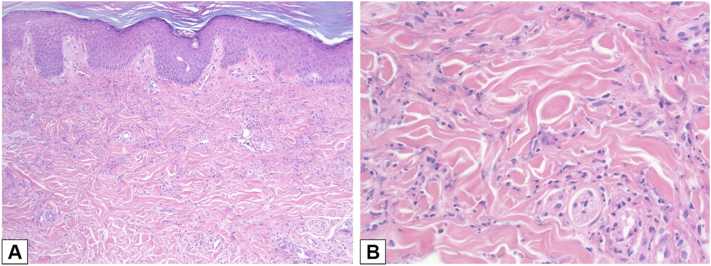
H&E of initial presentation of case 1 demonstrating dermal neutrophilic infiltrate at **(A)** 10× magnification and **(B)** 40× magnification. *H&E*, Hematoxylin and eosin.

A 32-year-old female with a history of SLE presented with 3 weeks of worsening diffuse joint pains, pleuritic chest pain, blurry vision, and chronic intractable headaches in the setting of medication noncompliance and new periodontal infection. She had not been taking her prescribed immunosuppressives for 6 weeks. Her gum infection was treated with antibiotics, workup for her pleuritic chest pain was unremarkable, and she was started on oral steroids, hydroxychloroquine (HCQ), and mycophenolate mofetil (MMF) with initial improvement in symptoms and decreasing anti-double stranded DNA levels. On hospital day 12, despite being on a consistent dose of systemic steroids, she developed new painless, nonpruritic papules and plaques on the bilateral palms (Fig 1, *A*), worsening arthralgias, and facial and tongue swelling without any clear triggers. Her Systemic Lupus Erythematosus Disease Activity Index 2000 (SLEDAI-2K) score was 18 (Table II). Punch biopsy of her palmar rash revealed a dermal neutrophilic infiltrate without papillary edema (Fig 2). The dosing of her systemic steroids was increased with improvement in her rash and arthralgias and resolution of angioedema. After symptoms improved, she left the hospital against medical advice.

**Table II:** Systemic lupus erythematosus disease activity index 2000 (SLEDAI-2K)∗ scores at the time of presentation

Sign/symptom (points)	Case#			
	1A	1B	2	3
Lupus headache (8)	x	x		
Arthritis (4)	x	x		x
Pyuria (4)		x		
Inflammatory-type rash (2)	x	x	x	x
Alopecia (2)			x	
Oral or nasal mucosal ulcers (2)		x		
Low complement (2)	x	x	x	
High DNA binding (2)	x	x		
Fever (1)		x		
Thrombocytopenia (1)				
Final score†	18	25	6	6

∗ SLEDAI-2K scores were calculated using clinical and laboratory values 30 days prior to presentation.

† Scores of 6 or greater are considered clinically important.

Subsequently, she was seen by outpatient rheumatology for SLE flare with rash as well as persistent SLE disease activity requiring chronic systemic steroids. Given prior biopsy-proven ND, she was started on dapsone and colchicine with good control of her rash.

She was then readmitted 3 months after her initial presentation with 3 days of sudden-onset fevers, widespread painful pruritic rash, and worsening arthralgias. SLEDAI-2K score at presentation was 25 (Table II). Physical examination was notable for widespread tender, edematous papules and plaques sparing the palms and soles (Fig 1, *B*) and several nasal ulcers. Punch biopsy demonstrated superficial, sparse perivascular neutrophils. Her MMF and HCQ were continued, dapsone and colchicine dosing was increased, and she was treated with antihistamines and an increased dose of systemic steroids with taper. At discharge, her rash was faint and had nearly resolved. Unfortunately, she was ultimately lost to follow-up.

## Case 2

A 28-year-old male with SLE and lupus nephritis presented with a 2-month history of arthralgias, facial rash, and intermittent facial swelling. The rash was painful, nonpruritic, and episodic, lasting 2-3 days at a time. Examination revealed edematous pink plaques on the right arm and left periorbital area and light mottling of the left flank. Biopsy of the right arm revealed mild, diffuse interstitial neutrophilic infiltrate. His SLEDAI-2K score was 6 (Table II), and his immunosuppressive regimen at the time included HCQ, MMF, prednisone, and colchicine 0.6 mg daily. He was treated with dapsone, antihistamines, and an increased dose of colchicine, with improvement. Due to recurrent episodes of angioedema, the patient was started on anifrolumab. He was subsequently transitioned from anifrolumab to voclosporin in the setting of worsening renal function, but developed a recurrence of his rash with involvement of the left arm and face. Anifrolumab was reintroduced, and since then he has not had any notable recurrences of his rash or angioedema.

## Case 3

A 48-year-old female with a past medical history of minimal change disease presented with a 1-month history of bilateral thigh rash, muscle weakness, and joint pain that initially resolved with a short course of oral steroids but recurred on discontinuation. Her SLEDAI-2K score was 6 (Table II). On examination, she had nonpainful, nonpruritic pink annular plaques on the bilateral anterior thighs (Fig 1, *D*). Shave biopsy revealed mixed dermal infiltrate of neutrophils and rare eosinophils. Workup was notable for positive antinuclear antibody, positive anti-double stranded DNA, and elevated c-reactive protein. She was diagnosed with SLE and treated with a prednisone taper followed by initiation of HCQ with improvement in her symptoms and rash. Although her rash initially recurred while starting HCQ, she has not had any notable recurrences since establishing a stable treatment regimen for her SLE.

## Discussion

Although SLE is uncommonly associated with neutrophilic infiltrates, we found that SLE-associated NDs are a clinically important phenomenon associated with several unifying features. Specifically, all patients had active SLE at the time of presentation, as evidenced by elevated SLEDAI-2K scores^8^ or systemic flares. Clinically, all patients developed erythematous to pink papules and plaques, which were often edematous and involved the extremities. Pruritus was absent or mild, and 2 patients developed associated angioedema. None of the 3 patients had a prior history of acute CLE, subacute CLE, or discoid lupus erythematosus. During the study period, no lesions were clinically or histologically consistent with these classic CLE subtypes. The neutrophilic dermatoses described herein were the predominant cutaneous manifestations observed. Treatment responses were favorable with escalation of systemic immunosuppression, and 2 patients required adjunctive agents such as dapsone, colchicine, or anifrolumab for complete disease control. These shared features support the concept that SLE-associated NDs are a marker of systemic disease activity and respond to therapies targeting both lupus and neutrophil-driven inflammation.

Unfortunately for diagnostic purposes, SLE-associated NDs are a heterogeneous group with diverse clinical and histopathologic presentations. In our small series, clinical presentation varied from localized to widespread lesions of varying morphologies, although the extremities were always involved, and edematous pink papules and plaques were always present. Similarly, prior studies investigating SLE-associated NDs have found no pathognomonic clinical findings; however, papules and plaques were the most common morphologies, and the extremities were often involved.^1^^,^^2^

The histopathologic differential diagnosis for the biopsies in our series included Sweet syndrome–like ND and NUD. The latter is characterized by an interstitial and perivascular neutrophilic infiltrate without significant papillary dermal edema. Given its reported association with connective tissue diseases including SLE, NUD should be considered when evaluating neutrophil-rich infiltrates in patients with systemic autoimmune disease.^9^^,^^10^ Our histopathologic findings were diverse, ranging from sparse, superficial infiltrates to more cellular, Sweet-like reactions. While the presence of interface changes and dermal mucin has been proposed as a histopathologic clue to diagnosis, prior work^1^^,^^2^ has shown that these are not sensitive for SLE-associated ND. In our 4 biopsies, none had interface changes.

SLE-associated NDs may be the presenting symptom of disease and can manifest in patients without prior diagnosis of SLE.^1^, ^2^, ^3^^,^^5^^,^^11^ Larson and Granter^1^ noted that in patients with undiagnosed SLE presenting with SLE-associated ND, histopathology did not commonly reveal background changes (ie, interface change, dermal mucin, and basement membrane zone thickening) that would provide an additional clue to the diagnosis of SLE. Thus, in the absence of histologic clues and prior diagnosis of SLE, clinical signs and symptoms of underlying systemic disease are key to an accurate differential diagnosis. For example, in one of our cases, a patient presented only with localized rash and vague systemic symptoms of muscle weakness and arthralgias. She was ultimately diagnosed with SLE. This illustrates the importance of conducting a thorough review of systems and of including cutaneous manifestations of SLE on the differential diagnosis of neutrophilic infiltrates within the correct clinical context.

The pathogenesis of SLE-associated NDs is unclear; however, it has been hypothesized that they may represent an antibody-antigen complex-mediated pathology.^2^^,^^4^ While it was previously proposed that ultraviolet exposure may drive the pathogenesis of SLE-associated NDs, this was based on a series by Pavlidaky et al^3^ observing a high proportion of photodistributed eruptions in patients with SLE-associated NDs. Additional studies^1^^,^^2^^,^^5^ have not noted a strong association. In our series, none of our patients had a photodistributed rash. In another series of autoimmunity-related ND, most patients used sunscreen and avoided the sun,^3^ so sun exposure was less likely to play a role.

In summary, this series highlights that SLE-associated neutrophilic dermatoses seem to be a clinically significant marker of systemic disease activity and can be treated by controlling the underlying systemic disease. SLE-associated NDs present with a wide range of clinical and histopathologic features, although all our cases had edematous pink papules and plaques involving the extremities. SLE-associated NDs may be the presenting sign of underlying undiagnosed SLE, so clinical correlation is paramount to prompt diagnosis, and the presence of neutrophilic infiltrates on histopathology should raise concern for SLE in context of other characteristic symptoms.

## Conflicts of interest

None disclosed.
